# OpenEvidence: Enhancing Medical Student Clinical Rotations With AI but With Limitations

**DOI:** 10.7759/cureus.76867

**Published:** 2025-01-03

**Authors:** Niket Patel, Harpreet Grewal, Venkata Buddhavarapu, Gagandeep Dhillon

**Affiliations:** 1 Medicine, Drexel University College of Medicine, Philadelphia, USA; 2 Radiology, Florida State University College of Medicine, Pensacola, USA; 3 Internal Medicine, Banner Health, Mesa, USA; 4 Internal Medicine, University of Maryland Medical Center, Glen Burnie, USA

**Keywords:** artificial intelligence (ai) in medicine, chat gpt, evidence-based clinical practice, medical education, medical student, openevidence

## Abstract

The integration of artificial intelligence (AI) into healthcare has introduced tools that improve medical education and clinical practice. OpenEvidence is an example, providing real-time synthesis and access to medical literature, particularly for medical students during clinical rotations. By enabling efficient searches for clinical guidelines, diagnostic criteria, and therapeutic approaches, it streamlines decision-making and study preparation. Its ability to present recent publications and highlight less commonly discussed treatments supports evidence-based learning. Despite these strengths, OpenEvidence has limitations. It struggles with targeted searches for specific articles, authors, or journals and operates through an opaque curation process. Compared to ChatGPT, which offers conversational interactivity, and UpToDate, known for its comprehensive, CME-accredited content, OpenEvidence lacks certain advanced features. However, its user-friendly design and focus on clinical evidence make it a valuable, accessible alternative. This editorial critically examines OpenEvidence's capabilities and limitations, comparing it with established tools. It emphasizes the need for greater transparency, broader evidence integration, and enhanced functionality to maximize its impact. Addressing these challenges could improve OpenEvidence's utility, supporting a more effective, evidence-based approach to medical education and clinical practice.

## Editorial

Introduction

The rapid advancement of artificial intelligence (AI) in healthcare has introduced tools like OpenEvidence, which aim to improve the accessibility and synthesis of medical literature [1,2]. Designed to assist medical students during clinical rotations, OpenEvidence provides evidence-based summaries, direct links to research articles, and up-to-date information on clinical guidelines, diagnostic criteria, and therapeutic approaches. These features make it a potentially valuable resource for decision-making and study preparation. However, OpenEvidence has notable limitations, including its inability to perform targeted searches for specific article titles, authors, or journals, and a lack of interactivity or comprehensive resources when compared to other tools like UpToDate or ChatGPT.

The role of OpenEvidence in clinical rotations

Access to reliable, evidence-based information is essential during clinical rotations, where students face diverse and challenging cases. OpenEvidence provides guidance on topics such as differential diagnoses, treatment protocols, and dosing recommendations. It highlights less commonly discussed treatments, such as buspirone for obsessive-compulsive disorder (OCD), doxycycline for recurrent aphthous stomatitis, and treatment options for rare diseases like Erdheim-Chester disease [3-5]. Additionally, OpenEvidence synthesizes diagnostic insights, such as radiological findings in Wilson's disease, including the "Face of the Giant Panda" and "Split Thalamus" signs [6]. These features aim to provide students with insights into both common and rare scenarios.

A unique feature of OpenEvidence is its "Featured" tab, which highlights articles selected by its team, including recent publications from 2024. This is complemented by "Trending" and "New Evidence" tabs within the "Feed" section, which can be filtered by medical specialties. The platform also facilitates quick access to clinically relevant content, enabling medical students to integrate learning with practice. Its versatility extends to crafting multiple-choice questions, creating tables, calculating risk scores, and writing patient handouts. These tools are designed to support application in clinical settings and facilitate learning during rotations.

For medical students, OpenEvidence’s user-friendly interface may integrate into workflows, helping them gather information on clinical presentations and physical exams efficiently, which is critical during demanding rotations where burnout is a risk [7]. The platform minimizes time spent navigating complex interfaces or lengthy articles, allowing students to focus on applying knowledge in patient care scenarios. By prioritizing the latest studies and clinical guidelines, OpenEvidence promotes evidence-based habits and enhances skill development.

OpenEvidence in context: strengths and limitations compared to ChatGPT and UpToDate

While OpenEvidence presents innovative features, it faces significant limitations that hinder its effectiveness. A major drawback is its inability to perform targeted searches for specific article titles, authors, or journals, challenging users seeking precise resources like landmark studies or guidelines from top journals. Unlike ChatGPT, an AI conversational platform designed for dynamic, interactive dialogue, OpenEvidence lacks advanced conversational abilities, limiting its capacity to help users clarify ambiguities or explore complex topics interactively [8]. Furthermore, a limitation becomes evident when examining OpenEvidence's ability to provide a deeper body of supporting evidence for certain treatments. For instance, while it identified buspirone’s use in OCD, it did not surface additional studies, such as its role as an adjuvant therapy in fluoxetine-treated patients, which might inspire greater confidence in its application to patient care [9]. This gap highlights the need for broader evidence aggregation to ensure more robust clinical recommendations. Additionally, the platform lacks visual aids, such as diagnostic imaging or interactive decision trees, which could enhance its utility for complex clinical scenarios. Its opaque curation process, with unclear criteria for selecting or prioritizing evidence, further reduces user confidence. Compared to UpToDate, a widely used clinical decision-support tool offering peer-reviewed, structured content and CME credits, OpenEvidence lacks features that support professional development, diminishing its appeal for healthcare practitioners seeking tools that enhance both learning and credentialing [10,11]. Addressing these weaknesses could transform OpenEvidence into a more comprehensive and versatile medical resource.

Despite these challenges, OpenEvidence has successfully addressed certain weaknesses present in ChatGPT. ChatGPT, while versatile and capable of facilitating dynamic, interactive dialogue across a broad range of topics, has notable limitations that undermine its utility for medical and academic purposes. One key issue is its tendency to fabricate information or generate nonexistent references, a phenomenon known as "AI hallucination" [12]. ChatGPT also relies on static training datasets, which often result in outdated responses for rapidly evolving medical topics. For example, it might suggest obsolete protocols for managing diseases like COVID-19, where guidelines frequently evolve. In contrast, OpenEvidence updates its database regularly and provides real-time, evidence-based answers. Sources are often marked with labels such as “New Research” for recent studies or “Leading Journal” for high-impact publications, enhancing both credibility and timeliness. This transparency may inspire greater user confidence compared to ChatGPT due to its inability to label sources in a similar way. OpenEvidence also retains prior user queries for convenient access, similar to ChatGPT, and is freely available to eligible medical students and healthcare professionals via a Gmail account. While ChatGPT’s conversational abilities allow for flexible exploration of topics, its reliance on static data and lack of specificity limit its utility for precise medical decision-making. OpenEvidence focuses on updated, credible sources to address evidence-based medical inquiries, even though it lacks ChatGPT’s capacity for interactive exploration.

OpenEvidence also addresses several limitations of UpToDate, a widely used clinical decision-support tool, while retaining some of its valuable features. UpToDate’s high subscription cost restricts access for students and smaller institutions in resource-constrained settings, whereas OpenEvidence offers free access to eligible users. In addition, UpToDate’s extensive content can overwhelm users seeking concise recommendations during urgent clinical scenarios. OpenEvidence attempts to address this with evidence summaries and specialty filters aimed at improving information retrieval. Moreover, while UpToDate focuses heavily on consensus-based Western medicine, potentially underrepresenting global or alternative practices, OpenEvidence’s continuous updates aim to provide a broader range of evidence. Notably, OpenEvidence mirrors UpToDate’s emphasis on peer-reviewed, clinically relevant content, ensuring its summaries remain trustworthy. Though it lacks UpToDate’s CME-accreditation features, OpenEvidence’s focus on accessibility, usability, and relevance offers an alternative for medical students and professionals.

Lastly, OpenEvidence shares limitations with both ChatGPT and UpToDate, particularly in its inability to create actionable recommendations tailored to specific patient scenarios. Like ChatGPT, OpenEvidence struggles to synthesize complex information into nuanced clinical decision-making tools. For example, it cannot effectively integrate a patient’s past medical history, physical examination findings, review of systems, and other pertinent information to generate a personalized, actionable care plan. This limitation reduces its utility for clinicians managing multifaceted cases that require individualized approaches. Similar to UpToDate, OpenEvidence presents evidence in static formats, lacking the interactive tools or visualization features, such as decision trees or diagnostic algorithms, that could enhance its applicability in dynamic clinical environments. Addressing these shared challenges, such as improving its ability to contextualize and personalize recommendations, would allow OpenEvidence to evolve into a more effective resource for real-world medical decision-making.

Practical and functional considerations

Building on the earlier discussion of transparency, the curation process in OpenEvidence continues to raise concerns, as users remain uncertain about how articles are selected, prioritized, or excluded. Providing clearer criteria, such as emphasizing recency, clinical relevance, or impact factor, and specifying whether decisions are made by experts or automated systems could enhance user trust. To address this further, a participatory feature allowing users to submit articles not currently in the database for review would be a valuable addition. Combined with a clear and robust vetting process, this approach could mitigate biases in source selection while broadening the platform’s scope. By taking these steps, OpenEvidence could reinforce its credibility and expand its utility within medical education and clinical practice [13].

From a technical perspective, OpenEvidence has limitations that can disrupt its usability. Persistent bugs, such as requiring users to re-enter prompts without a clear indication of what went wrong, hinder the platform’s ability to provide a seamless experience. Errors generated when evidence is insufficient, though somewhat informative, disrupt the user experience. Proactive suggestions for refining prompts or providing alternative resources could mitigate these interruptions. Furthermore, the inability to display medical images, even though it can describe them, limits OpenEvidence's usefulness for visual diagnoses and related educational tasks [14]. This gap underscores the importance of integrating visual resources to enhance its textual evidence.

The platform’s opaque methodology for processing and prioritizing information compounds concerns about its reliability, particularly for users wary of relying on AI tools functioning as “black boxes” [15]. When users submit prompts, the criteria for selecting specific sources remain unclear. Previously mentioned tags like “New Research” or “Leading Journal” occasionally provide some insight, but the rationale behind these labels and their inconsistent application is ambiguous. In addition, identical prompts can sometimes yield differently worded responses or varying sources, which may reduce user trust and confidence in the platform’s consistency.

The “Feed” feature, particularly its “Featured” subsection, requires improvement. Featured queries, which are also displayed on the homepage, occasionally lead to a blank prompt, sometimes even with the first recommended query. These glitches reduce the platform’s reliability and undermine its goal of offering ready-to-use queries.

By addressing these technical shortcomings, OpenEvidence could significantly bolster its reliability, usability, and overall user satisfaction.

Ethical and educational implications

The integration of AI into medical practice raises ethical concerns, particularly regarding the risk of over-reliance on tools like OpenEvidence, which could diminish critical thinking skills essential for clinical practice [16]. Inconsistencies or errors in AI-generated results emphasize the need for human oversight to ensure accurate interpretation and adaptability to complex scenarios [17]. While OpenEvidence supports evidence-based learning during rotations, its inability to generate nuanced, actionable recommendations tailored to specific patient cases limits its educational potential. Clear guidelines emphasizing AI as a supplement, not a substitute for clinical judgment, could strengthen OpenEvidence’s role as a secondary resource in medical education [18].

Conclusion: a valuable but evolving tool

OpenEvidence has the potential to offer considerable value for medical students during clinical rotations by providing timely, evidence-based information that bridges the gap between learning and real-world application. Its accessibility and practical features position it as a strong contender among existing platforms. However, its full potential remains constrained by unique shortcomings and challenges it shares with other tools in this space.

To become a more comprehensive resource, OpenEvidence must address transparency concerns by clarifying its curation process, particularly the criteria used for selecting and prioritizing sources. Clearer labeling of evidence and a participatory feature for user-submitted article uploads could improve its credibility and foster trust. Furthermore, resolving technical issues, such as glitches in the "Feed" and inconsistencies in source selection, is essential. OpenEvidence must also address shared limitations with ChatGPT and UpToDate, such as the inability to synthesize complex patient data into actionable clinical plans and the lack of interactive decision-making tools while continuing efforts to achieve CME accreditation.

Despite these challenges, OpenEvidence’s evolving features and commitment to evidence-based practice position it as a promising supplement to existing resources. By addressing its own limitations and those shared with other platforms, it has the potential to become an indispensable tool for medical education and clinical care, equipping future healthcare professionals to navigate an increasingly AI-integrated healthcare environment with precision and confidence.
